# Preoperative smoking cessation interventions: a systematic review and meta-analysis

**DOI:** 10.1186/s13741-024-00479-4

**Published:** 2025-01-10

**Authors:** Mohammed Alsanad, Mohammed Aljanoubi, Faraj K. Alenezi, Amanda Farley, Babu Naidu, Joyce Yeung

**Affiliations:** 1 College of Applied Medical Sciences, King Saud Bin Abdulaziz University for Health Sciences, Jeddah, Saudi Arabia; 2 Warwick Clinical Trials Unit, Warwick Medical School, University of Warwick, Coventry, UK; 3 King Abdullah International Medical Research Center, Jeddah, Saudi Arabia; 4 College of Applied Medical Sciences, King Saud Bin Abdulaziz University for Health Sciences, Riyadh, Saudi Arabia; 5 King Abdullah International Medical Research Center, Riyadh, Saudi Arabia; 6 Institute of Applied Health Research, University of Birmingham, Birmingham, UK; 7 Thoracic Surgery, University Hospitals Birmingham NHS Foundation Trust, Birmingham, UK; 8 Birmingham Acute Care Research Group, Institute of Inflammation and Ageing, College of Medical and Dental Sciences, University of Birmingham, Birmingham, UK; 9 Critical Care Medicine, University Hospitals Birmingham NHS Foundation Trust, Birmingham, UK

**Keywords:** Preoperative smoking cessation interventions, Systematic review, Post-operative complications, Meta-analysis

## Abstract

**Background:**

Smoking is the leading single cause of preventable death in England and also increases the risk of postoperative complications. The preoperative period is a potential opportunity to introduce smoking cessation interventions to smokers to reduce the risk of postoperative complications. A systematic search was conducted to find all studies that investigated the effectiveness of preoperative smoking cessation interventions. The primary outcome was smoking cessation at surgical time to the last follow-up, and the secondary outcome was postoperative complications that required treatment or ICU admission. A random-effects meta-analysis was used to synthesize the outcomes. Sixteen studies were included in the review (3505 participants), and 14 studies were included in the meta-analysis (2940 randomized participants). The quality of evidence was moderate due to the high risk of bias and heterogeneity. We found that patients who were provided with a smoking cessation intervention had significantly increased odds of quitting smoking by the time of surgery compared with usual care, with a reported relative risk (95% CI) 1.64 (1.30–2.07) and at the longest follow-ups with RR (95% CI) 1.38 (1.12–1.70). Moreover, there was no difference found in the rate of postoperative complications between intervention and control conditions with RR (95% CI) 0.81 (0.62–1.06). The use of standardized outcome measurements is recommended to reduce heterogeneity for future studies, and further investigation focusing on patient perspectives is needed.

**Trial registration:**

PROSPERO CRD42023423202.

**Supplementary Information:**

The online version contains supplementary material available at 10.1186/s13741-024-00479-4.

## Introduction

There were approximately 5.4 million smokers in England in 2019 (Office for National Statistics 2023). The prevalence of smoking reduced from 19.8% in 2011 to 13.9 in 2019, and now, in 2024, the smoking prevalence in England has reached 12.7%. However, tobacco smoking is still the leading single cause of preventable death in England causing an estimated more than 76,000 deaths per year (Action on Smoking and Health 2023). According to Cancer Research UK, 72% of lung cancers are caused by smoking (CRUK 2018). It has been reported that smokers who undergo lung cancer surgery have more severe pain (Action on Smoking and Health 2021), lower quality of life (Doll et al. 2004), longer hospital stays (Halpern et al. 1993), a higher risk of postoperative complications (Burnham 2023), and a higher death rate than non-smokers (Khan 2022). Smoking cessation at any age or stage would significantly reduce the risk of lung cancer (Doll et al. 2004; Halpern et al. 1993), and nearly 60% of smokers wish to quit,however, 95% of unsupported quit attempts result in a relapse within a year (Burnham 2023).

According to the current National Institute for Health and Care Excellence (NICE) recommendations, smokers having elective surgery should receive behavioral counseling and medication to stop smoking as soon as possible during their outpatient or preoperative evaluations (National Institute for Health and Care Excellence 2021). The recommendation is a weekly session for a minimum of 4 weeks after the quit date, in person if possible (National Institute for Health and Care Excellence 2021). The preoperative period is a golden opportunity to introduce smoking cessation interventions to smokers who usually are at high risk of complications. Patients scheduled for surgery tend to be motivated and have a positive attitude toward improving their health behaviors, including quitting smoking (Shi and Warner 2010). However, despite this opportunity, the NICE recommendations, and the critical complication of smoking for surgical patients, the majority of smokers going for thoracic surgery do not receive preoperative smoking cessation support. For example, 40% of smokers were offered smoking cessation interventions in a UK thoracic unit in 2015 (Webb et al. 2015), and only 30% of patients going for thoracic cancer surgery reported abstinence at the time of surgery (Lugg et al. 2017). In a more recent audit done by the British Thoracic Society (BTS) on smoking status and prevalence, they reported that 77% of surgical patients were recorded as smokers, and 21% of these patients were current smokers.

This systematic review aims to investigate the impact of preoperative smoking cessation interventions on smoking abstinence and on the continuous quit rate and the reported incidence of any postoperative complications. By synthesizing current evidence and evaluating the potential of smoking cessation interventions, this review seeks to inform clinical guidelines and improve patient outcomes, with a broader impact on healthcare resource optimization.

## Method

Literature searches were conducted according to the Cochrane Handbook for Systematic Reviews of Interventions (Higgins et al. 2023). We used the following PICO (Population, Intervention, Comparator, Outcome) framework to construct our review question. We included all individuals of both sexes aged over 18 who were current smokers and were scheduled for an elective surgical procedure (P). Studies that recruited only pregnant women were excluded. We included randomized control trials (RCTs) and non-RCTs conducted on preoperative smoking cessation interventions (I). We excluded abstracts, grey literature, and unpublished data with no language limitation. Preoperative smoking cessation intervention was defined as any smoking cessation support, that is, one or two types of intervention that were initiated at least 48 h before surgery and that may or may not have continued after surgery with follow-up to at least 3 months after surgery. The control groups were the surgical patients who were advised to quit smoking preoperatively and given the usual care (C). The primary outcome was smoking cessation at surgery time to the last follow-up (O). Smoking or tobacco smoking was defined as the use of any smoking tobacco products, including cigars, cigarettes, pipes, and any other form of tobacco inhalation. All forms of smoking abstinence were included. When studies were conducted on mixed cohorts including smokers and non-smokers or surgical and non-surgical patients, these studies were included if more than 80% met the inclusion criteria, the smokers in their studies were reported as a subgroup, and/or smoking cessation was a primary or secondary outcome.

The primary outcome for meta-analysis was continuous smoking cessation at the time of surgery, and the co-primary outcome was continuous smoking cessation at the last follow-up using either a biochemically validated or unvalidated self-reported abstinence. Smoking cessation at the time of surgery was defined as smoking abstinence before the surgery or within 30 days of the procedure. The secondary outcomes were postoperative pulmonary complications and other major postoperative complications that require treatment or ICU admission using the Postoperative Morbidity Survey (POMS) (Bennett-Guerrero et al. 1999). We searched Medline, EMBASE, Web of Science, the Cumulative Index to Nursing and Allied Health Literature (CINAHL), and Cochrane Library from inception to 1/12/2023 without any filter or limits. The search strategy process was assisted and validated by an academic librarian and was reported according to the Preferred Reporting Items for Systematic Reviews and Meta-Analyses Statements (PRISMA-S) protocol (Moher et al. 2015). A sample search strategy was utilized using subject headings including “preoperative”, “smoking”, and “cessation”, along with other related text words (Appendix 1).

For the purpose of data management and duplicate removal, all entries from the five databases were imported into the EndNote (Version 20) program (Clarivate n.d.). After the duplicates were removed, the remaining data were imported into Rayyan, a web tool designed for systematic and scoping reviews (Ouzzani et al. 2016). Using the predetermined inclusion and exclusion criteria and the PICO framework, two independent reviewers (MS and MJ) blindly evaluated the relevancy of the titles and abstracts. After each screening stage, a meeting was conducted between the reviewers to resolve any disagreement on the relevance of the articles. After this step, when the reviewers had agreed on the relevance of the articles, the full text was acquired, exported to Endnote, and screened for eligibility. The full-text screening was conducted on all studies that matched the predetermined criteria. All the data were screened by both reviewers, and Cohen’s kappa was used to measure interrater reliability and degree of accuracy in a statistical classification. A third reviewer (JY) was contacted to resolve any conflict. The data-extracting form was developed using a Google Form, and then the information was imported into a Microsoft Excel sheet (version 16.82) (Appendix 2). MS and MJ piloted the comprehensiveness of the extraction form by randomly selecting four studies,a comparison was then made between them to identify possible differences. All the required adjustments to the form were made. The final extraction form was used to obtain the information needed by MS and MJ. When the information was unclear or missing, the authors were contacted for more details and for clarification. Any conflict was resolved by the third reviewer (JY).

A descriptive analysis was performed of the included studies’ demographic data, methodologies, intervention and comparator details, and outcome. RevMan (version: 7.9.0, 2024) was chosen for the meta-analyses using the Haenszel random effect and for presenting the data using forest plot figures with a risk of bias assessment (Review Manager Web (RevMan Web) 2024). Meta-analyses were conducted on smoking cessation at the time of surgery, at the last follow-up, and on any postoperative complications. Risk ratios (RR) and a 95% confidence interval (95%CI) were used for smoking cessation and postoperative complications using intention to treat and available-case analysis according to the Cochrane Tobacco Addiction Group (TAG) recommendations (Cochrane Tobacco Addiction T 2022). We used the number of patients randomized as the denominators, excluding patients who died before follow-up, for whom surgeries were canceled or postponed, or who withdrew before receiving the intervention. All participants lost to follow-up were assumed to be smoking in line with the Russell standard definition of smoking abstinence in RCTs (West et al. 2005). The Cochrane Risk of Bias 2 (ROB 2.0) tool was used to assess the risk of bias of the RCTs (Sterne et al. 2019) (Appendix 3), the National Institution of Health (NIH) Quality Assessment Tool for Before-After (Pre-Post) Studies With No Control Group for pre-post studies, and the NIH Quality Assessment Tool for Observational Cohort and Cross-Sectional Studies for observational studies (National Institutes of Health N 2013) (Appendix 4). Two independent reviewers assessed the risk of bias and quality of the studies independently. The *I*^2^ statistic was used to assess heterogeneity. We conducted a sensitivity analysis excluding a pilot study and studies with > 20% dropout to assess the effect of missing data. We performed a subgroup analysis by pooling studies according to the intensity of the behavioral support (intensive and brief behavioral support) and according to the length of follow-up (3–6 months and > 6 months). This review was prospectively registered in PROSPERO (CRD42023423202).

## Result

**Fig. 1:**
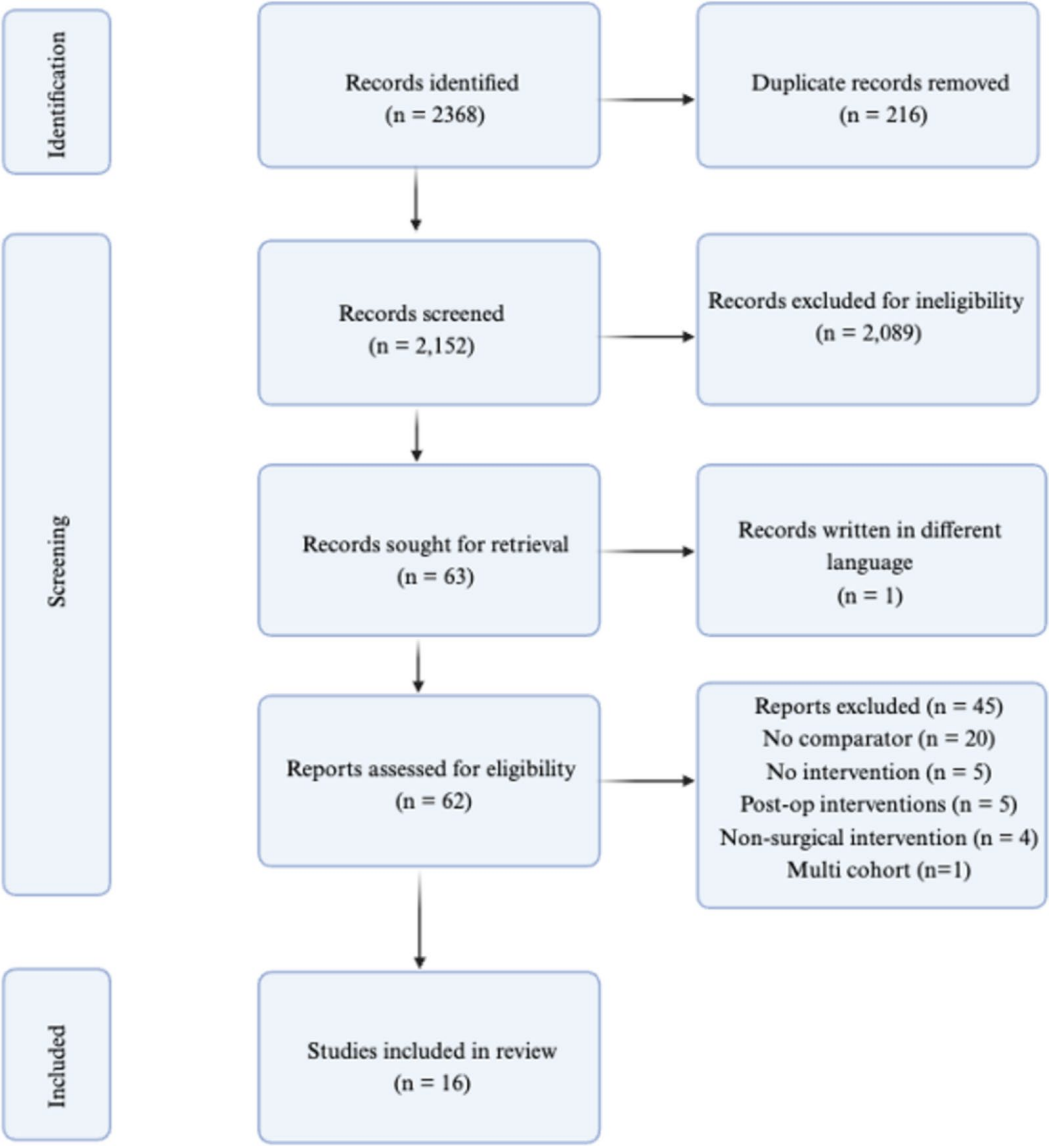
PRISMA diagram

The initial search identified 2368 studies. Endnote identified 216 duplicates and removed them automatically. The remaining 2152 studies were reviewed by titles and abstracts. After removing the studies due to ineligibility, 62 studies were reviewed in full text. Sixteen studies were included in the systematic review (Alba et al. 2022; Beaupre et al. 2019; Lauridsen et al. 2022; Lee et al. 2013; Møller et al. 2002; Ostroff et al. 2014; Ratner et al. 2004; Rojewski et al. 2021; Lindström et al. 2008; Thomsen et al. 2010; Warner et al. 2011; Webb et al. 2014; Webb et al. 2022; Wolfenden et al. 2005; Wong et al. 2017; Wong et al. 2012). The details of the screening process are reported in the PRISMA diagram (Fig. 1). Sixteen studies were included in this review, with 14 incorporated into the meta-analysis, involving 2940 randomized participants (Tables 1 and 2). Two studies were excluded from the meta-analysis, as they were not RCTs (Beaupre et al. 2019; Webb et al. 2014). Four studies reported more than 20% loss to follow-up (Ratner et al. 2004; Rojewski et al. 2021; Warner et al. 2011; Webb et al. 2022). All the studies included in the meta-analysis examined the primary outcome, while seven examined the secondary outcome. Eight studies were within the last 10 years. Most of the studies were from developed countries. The overall mean age for all participants was 50.4 years. Nine studies included mixed elective surgery (Alba et al. 2022; Lee et al. 2013; Ratner et al. 2004; Warner et al. 2011; Webb et al. 2014; Webb et al. 2022; Wolfenden et al. 2005; Wong et al. 2017; Wong et al. 2012) with three of these excluding cardiac surgery (Wolfenden et al. 2005; Wong et al. 2017; Wong et al. 2012) and one excluding cardiac and neurosurgery (Webb et al. 2022). Moreover, the other studies were more focused on specific surgeries such as elective orthopedic surgery (Beaupre et al. 2019; Møller et al. 2002), mixed cancer surgery (Ostroff et al. 2014; Rojewski et al. 2021), breast cancer (Thomsen et al. 2010), both general and orthopedic surgery (Lindström et al. 2008), and radical cystectomy for bladder cancer (Lauridsen et al. 2022). All the interventions in the included studies used two or more types of smoking cessation support, such as pharmacotherapy with behavioral support. Usual care is defined as referring patients to the smoking cessation support provided at a local pharmacy, family doctor, or smoking cessation services in the study’s country. Any extra support to the comparator is mentioned in Tables 1 and 2 along with all the study details. Two studies were classified as intensive intervention, as they conducted multiple face-to-face behavioral support sessions at least 4 weeks before surgery with pharmacotherapy, while other studies used fewer sessions, started near the surgery time, or used one type of smoking cessation support. Most of the preoperative smoking cessation interventions in the included studies were delivered by clinicians. Most of the studies were considered as having a high risk of bias or with some concerns, and this was mainly due to missing data (Appendix 3). The Kappa score was 0.78 in title and abstract screening and 0.86 in the full screening,this means that the agreement percentage was more than 98%, which indicates substantial to almost perfect agreement and, therefore, ensures the consistency and clarity of the screening process (McHugh 2012).

**Table 1:** Characteristics of the 14 preoperative smoking cessation randomized control trials (RCT) included

	Authors	Year and country	Overall mean age	Type of surgery	Primary	Secondary	First measure	Last measure	Intervention	Control	Intervention delivered by
1	Alba et al. (2022)	2022 Colombia	41.8	Elective mix surgery	Behavioral change stage	Smoking cessation at 1 and 3 months	Surgery time	3 months	Behavioral support (2 sessions 5–10 min) by a family physician using 5 As F2F + telephone	Educational intervention By nurse	Clinician (doctors, nurses, specialist, etc.)
2	Lauridsen et al. (2022)	2022 Denmark	65.5	Radical cystectomy for bladder cancer	Postoperative complication	• Post-operative complications within 30 and 90 Smoking cessation and alcohol cessation up to 12 months postoperatively	Surgery time	1 year	Behavioral support (5 sessions) by a registered trained nurse Pharmacotherapy(chlordiazepoxide and NRT) Standard care F2F	Usual care	Research nurse
3	Lee et al. (2013)	2013 Canada	47.5	Elective mix surgery	Smoking cessation 7 days before surgery	• Post-operative complication Smoking cessation at 12 months	Surgery time	1 year	Behavioral support (1 brief, 4 via helpline with pharmacist) Pharmacotherapy (NRT) Brochures	Usual care	Clinician (doctors, nurses, specialist, etc.)
8	Lindström et al. (2008)	2008 Sweden	56.3	General and orthopaedic surgery	Peri-operative smoking cessation on the risk of postoperative complications	Short- and long-term smoking cessation	Surgery time	1 year	Behavioral support (4 sessions by nurse) Pharmacotherapy (NRT) F2F + telephone	Usual care	Clinician (doctors, nurses, specialist, etc.); professional in smoking cessation therapy
4	Møller et al. (2002)	2002 Denmark	65	Elective orthopaedic	Post-operative complication	Smoking cessation at 12 months	Surgery time	1 year	Behavioral support (6–8 sessions) by project trained nurse Pharmacotherapy (NRT) F2F	Usual care	Research nurse
5	Ostroff et al. (2014)	2014 USA	55.9	Mix cancer surgery	Preoperative smoking cessation, 3 months, 6 months	–	Surgery time	6 months	Behavioral support (5 sessions) Pharmacotherapy (NRT) Scheduled Reduced Smoking via app	Usual care	Clinician (doctors, nurses, specialist. etc.) and tobacco treatment specialists
6	Ratner et al. (2004)	2004 Canada	49.7	Elective mix surgery	Smoking cessation before surgery and 1 year abstinence in the postoperative period	–	Surgery time	1 year	Behavioural support (11 sessions) by registered nurse Pharmacotherapy (bupropion hydrochloride and NRT) F2F (2) + telephone (9)	Usual care	Clinician (doctors, nurses, specialist, etc.)
7	Rojewski et al. (2021)	2021 USA	56.9	Mix cancer surgery	Smoking cessation at surgery time	–	Surgery time	3 months	Behavioural support (2–5 sessions by in hospital-based tobacco treatment program) Pharmacotherapy (NRT) Monetary incentives contingent on abstinence F2F	Usual care (behavioural support + NRT)	Clinician (doctors, nurses, specialist, etc.); smoking cessation practitioner; pharmacist (out hospital); advanced practice registered nurse, clinical pharmacist, or psychologist from the hospital-based tobacco treatment program
9	Thomsen et al. (2010)	2010 Denmark	57	Breast cancer	Post-operative complications	Peri-operative and long-term smoking cessation	Surgery time	1 year	Behavioral support (one session 45–90 min) trained smoking cessation counselors Pharmacotherapy (NRT) F2F	Usual care	Smoking cessation practitioner; trained smoking cessation counselors
10	Warner et al. (2011)	2011 USA	49.2	Elective mix surgery	Rate of Quitline use	Smoking cessation at 30 and 90 days postoperatively	Surgery time	3 months	Brief advice Description of Quitline service Brochure with dedicated Quitline number	5 As approach	Clinician (doctors, nurses, specialist, etc.)
11	Webb et al. (2022)	2022 Australia	49.9	Non-cardiac and non-neuro Elective mix surgery	Smoking cessation at surgery time	Abstinence 3 months after surgery for those who had quit	Surgery time	3 months	Pharmacotherapy (NRT) Written information (brochure, info about NRT)	Usual care	Clinician (doctors, nurses, specialist, etc.)
12	Wolfenden et al. (2005)	2005 Australia	43.18	Non-cardiac Elective mix surgery	Smoking cessation at surgery and 3 months after attendance at a pre-op clinic	The cost of treatment/intervention delivery	Surgery time	3 months	Brief Advice Pharmacotherapy (NRT) Tailored counseling via a computer by research assistant Telephone and computer	Usual care	Clinician (doctors, nurses, specialist, etc.)
13	Wong et al. (2017)	2017 Canada	51.9	Non-cardiac elective mix surgery	Smoking cessation at 12 months	- Smoking cessation at 1, 3, and 6 months - Postoperative complication	Surgery time	1 year	Behavioral support (one 10–15 min session + Quitline sessions) done by anaesthesiologists and pharmacists Pharmacotherapy (3-month supply of varenicline) Educational pamphlet Fax referral to a Quitline for proactive telephone counseling	Behavioral support by research coordinator Placebo Standard materials	Research coordinators
14	Wong et al. (2012)	2012 Canada	52.6	Non-cardiac elective mix surgery	Smoking cessation at 12 months	- Smoking cessation at 3 and 6 months - Postoperative complication	Surgery time	1 year	Behavioral support by research coordinator Pharmacotherapy (Varenicline) Supplemented with standard printed materials F2F	Brief advice	Clinician (doctors, nurses, specialist, etc.); pharmacist (out hospital)

**Table 2:** Characteristics of the 2 other preoperative smoking cessation studies

	Authors	Year And country	Agee (mean)	Type of surgery	Primary	Secondary	Method	Intervention	Control	Intervention delivered by
15	Beaupre et al. (2019)	2019 Canada	58.7	elective orthopaedic surgery	Smoking cessation at surgery time	Use of the smoking cessation program	A pre-post-quasi-experimental study	Behavioral support by pharmacists Pharmacotherapy (patient’s preference) Educational video F2F	Usual care	Pharmacist (out of hospital); video
16	Webb et al. (2014)	2014 Australia	45.4	Elective mix surgery	Smoking cessation at surgery time	–	Questionnaire-based survey before and after intervention	Brief Advice Quitline referral form Educational brochure smoking cessation advice	Usual care	Clinician (doctors, nurses, specialist, etc.)

## Primary outcome

### Smoking cessation at the time of surgery

**Fig. 2:**
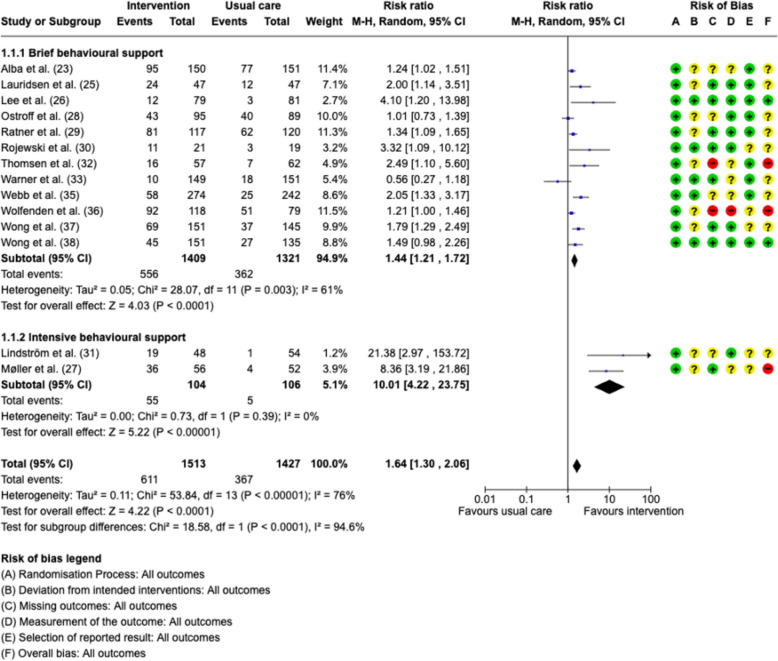
Forest plots for smoking cessation at the time of surgery. MH—Mantel–Haenszel random effects model. Risk of bias domains: **A** randomization process, **B** deviations from intended interventions, **C** missing outcomes, **D** measurement of the outcome, **E** selection of the reported result, **F** overall bias. Green circle—low risk of bias; yellow circle—some concerns about bias, red circle—high risk of bias

Compared to usual care, patients receiving a smoking cessation intervention had significantly increased odds of quitting smoking at the time of surgery, with a reported RR of 1.64 (95%CI 1.30–2.07, *p* < 0.0001), and the number needed to treat (NNT) was determined to be 7 (Fig. 2). The studies exhibited substantial heterogeneity (*I*2 = 76%).

Sensitivity analyses were conducted by excluding one pilot study; this resulted in a slightly lower RR of 1.60 (95%CI 1.27–2.01, *p* < 0.0001), with no difference in heterogeneity (*I*2 = 76%) (Rojewski et al. 2021). Further analysis, excluding studies with a dropout rate greater than 20%, showed an increased effect with an estimated RR of 1.78 (95%CI 1.33–2.40, *p* < 0.0001) (Ratner et al. 2004; Rojewski et al. 2021; Warner et al. 2011; Webb et al. 2022).

The meta-analysis also compared the effect of intervention intensity. Two studies providing high-intensity behavioral support resulted in an RR of 10.01 (95%CI 4.22–23.75, *p* < 0.0001) with no evidence of heterogeneity (Møller et al. 2002; Lindström et al. 2008). In contrast, the pooled estimate from 12 studies with less intensive support was an RR of 1.44 (95%CI 1.21–1.72, *p* < 0.0001), but with substantial heterogeneity (*I*2 = 61%). There was a statistically significant subgroup difference in smoking cessation at the time of surgery comparing intense and brief behavioral support (chi2 = 18.58, df = 1, *p* < 0.0001).

## Co-primary outcome

### Continuous smoking cessation at the last follow-up

**Fig. 3:**
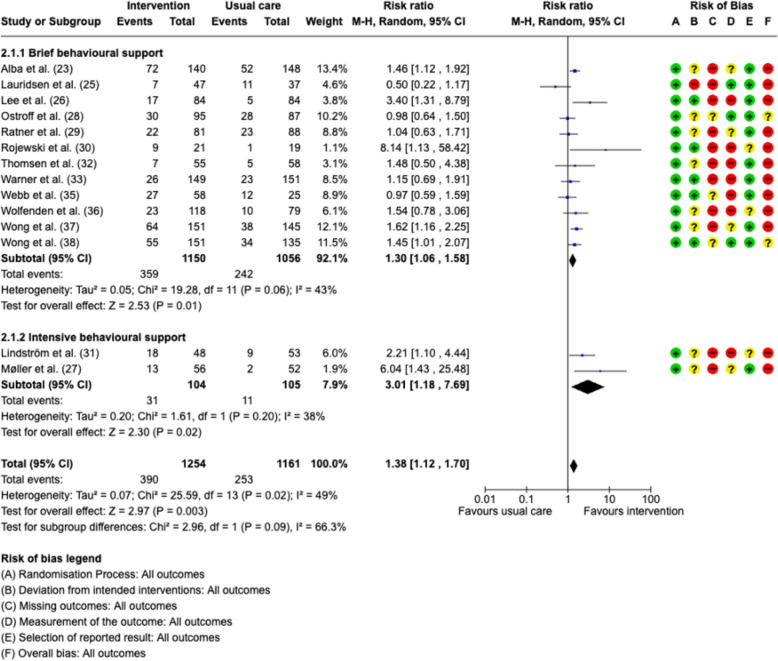
Forest plots for smoking cessation at the last follow-up. MH—Mantel–Haenszel random effects model. Risk of bias domains: **A** randomization process, **B** deviations from intended interventions, **C** missing outcomes, **D** measurement of the outcome, **E** selection of the reported result, **F** overall bias. Green circle—low risk of bias; yellow circle—some concerns about bias, red circle—high risk of bias

Fourteen studies assessed continuous smoking cessation after surgery (Alba et al. 2022; Lauridsen et al. 2022; Lee et al. 2013; Møller et al. 2002; Ostroff et al. 2014; Ratner et al. 2004; Rojewski et al. 2021; Lindström et al. 2008; Thomsen et al. 2010; Warner et al. 2011; Webb et al. 2022; Wolfenden et al. 2005; Wong et al. 2017; Wong et al. 2012) (Fig. 3). The meta-analysis revealed that preoperative smoking cessation interventions significantly increase the odds of quitting at the longest follow-up, with an RR of 1.38 (95%CI 1.12–1.70, *p* = 0.003), with moderate heterogeneity (*I*2 = 49%). The number needed to treat is 11. A pilot study was excluded by sensitivity analysis and showed a similar result with an RR of 1.35 (95%CI 1.10, 1.65, *p* = 0.004) with less heterogeneity (*I*2 = 46%) (Rojewski et al. 2021). A sensitivity analysis excluding four studies with brief behavioral support due to more than 20% dropout gave an overall effect estimate of RR of 1.48 (95%CI 1.16–1.80, *p* = 0.002), with moderate heterogeneity (*I*2 = 50%).

Two studies were pooled into an intensive behavioral support group, resulting in an RR of 3.01 (95%CI 1.18–7.69, *p* = 0.002) with low heterogeneity (*I*2 = 38%) (Møller et al. 2002; Lindström et al. 2008). The pooled estimate for the 12 less intensive studies was an RR of 1.30 (95%CI 1.06–1.58, *p* = 0.01), with moderate heterogeneity (*I*2 = 43%) (Alba et al. 2022; Lauridsen et al. 2022; Lee et al. 2013; Ostroff et al. 2014; Thomsen et al. 2010; Warner et al. 2011; Webb et al. 2022; Wolfenden et al. 2005; Wong et al. 2017; Wong et al. 2012). However, subgroup differences showed a chi2 of 2.96, df = 1 (*p* = 0.09), which indicated no significant differences between the intensive and the brief behavioral support groups.

## Secondary outcomes

### Postoperative complications

**Fig. 4:**
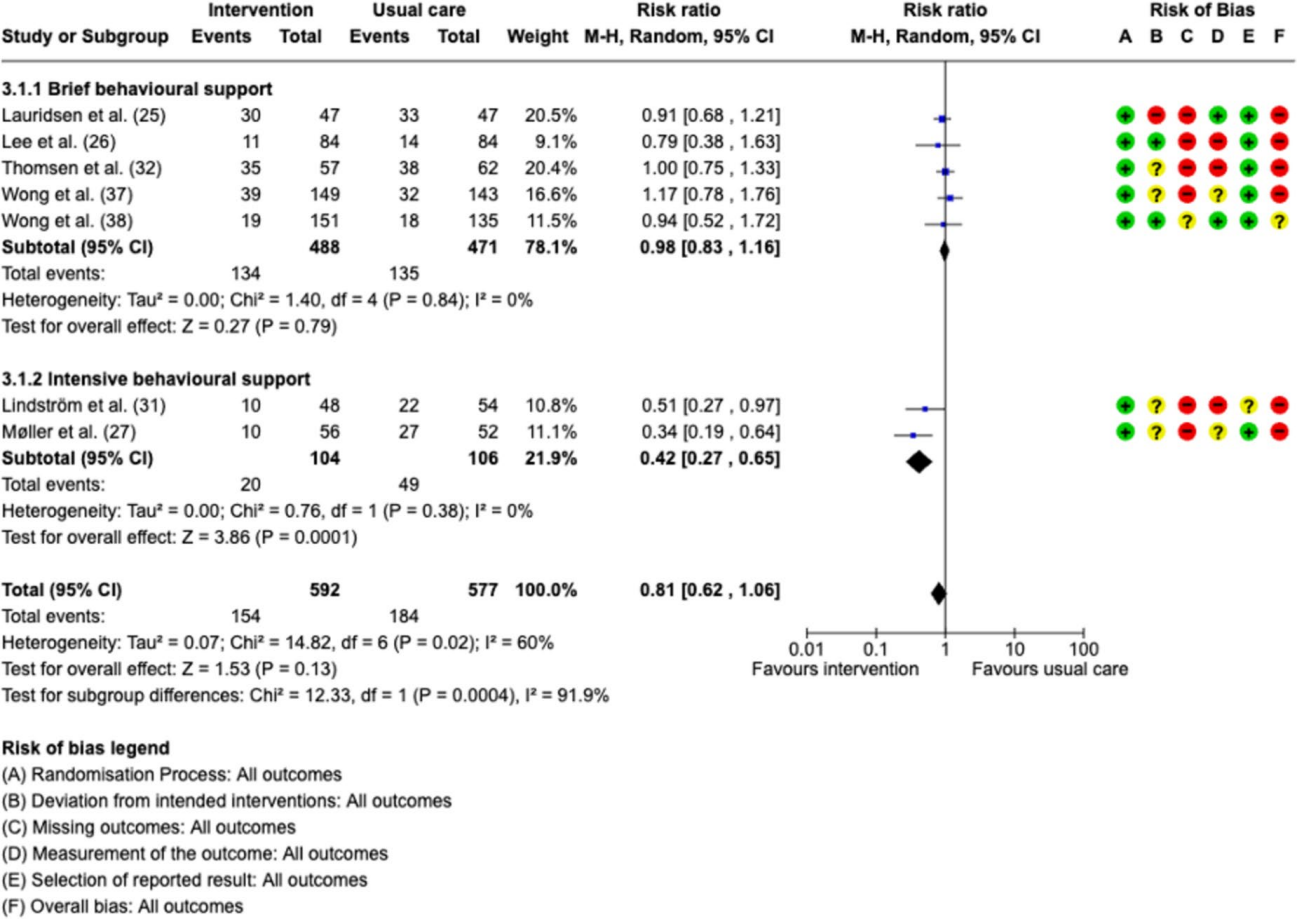
Forest plots for the incidence of postoperative complication. MH—Mantel–Haenszel random effects model. Risk of bias domains: **A** randomization process. **B** deviations from intended interventions, **C** missing outcomes, **D** measurement of the outcome, **E** selection of the reported result, **F** overall bias. Green circle—low risk of bias, yellow circle—some concerns about bias, red circle—high risk of bias

Seven studies reported postoperative complications (Lauridsen et al. 2022; Lee et al. 2013; Møller et al. 2002; Lindström et al. 2008; Thomsen et al. 2010; Wong et al. 2017; Wong et al. 2012). The overall effect across all smoking cessation interventions did not reduce postoperative complications, with an RR of 0.81 (95%CI 0.62–1.06, *p* = 0.13 (Fig. 4). Low-intensity behavioral support did not have a statistically significant effect on reducing post-operative complications with an RR of 0.98 (95%CI 0.83–1.16, *p* = 0.79) with no heterogeneity among the studies (*I*2 = 0%) (Lauridsen et al. 2022; Lee et al. 2013; Thomsen et al. 2010; Wong et al. 2017; Wong et al. 2012). On the other hand, there was a statistically significant reduction in postoperative complications in the high-intensity smoking cessation interventions group with an RR of 0.42 (95%CI 0.27–0.65, *p* = 0.0001) with no heterogeneity (*I*2 = 0%) (Møller et al. 2002; Lindström et al. 2008).

### Follow-up duration

**Fig. 5:**
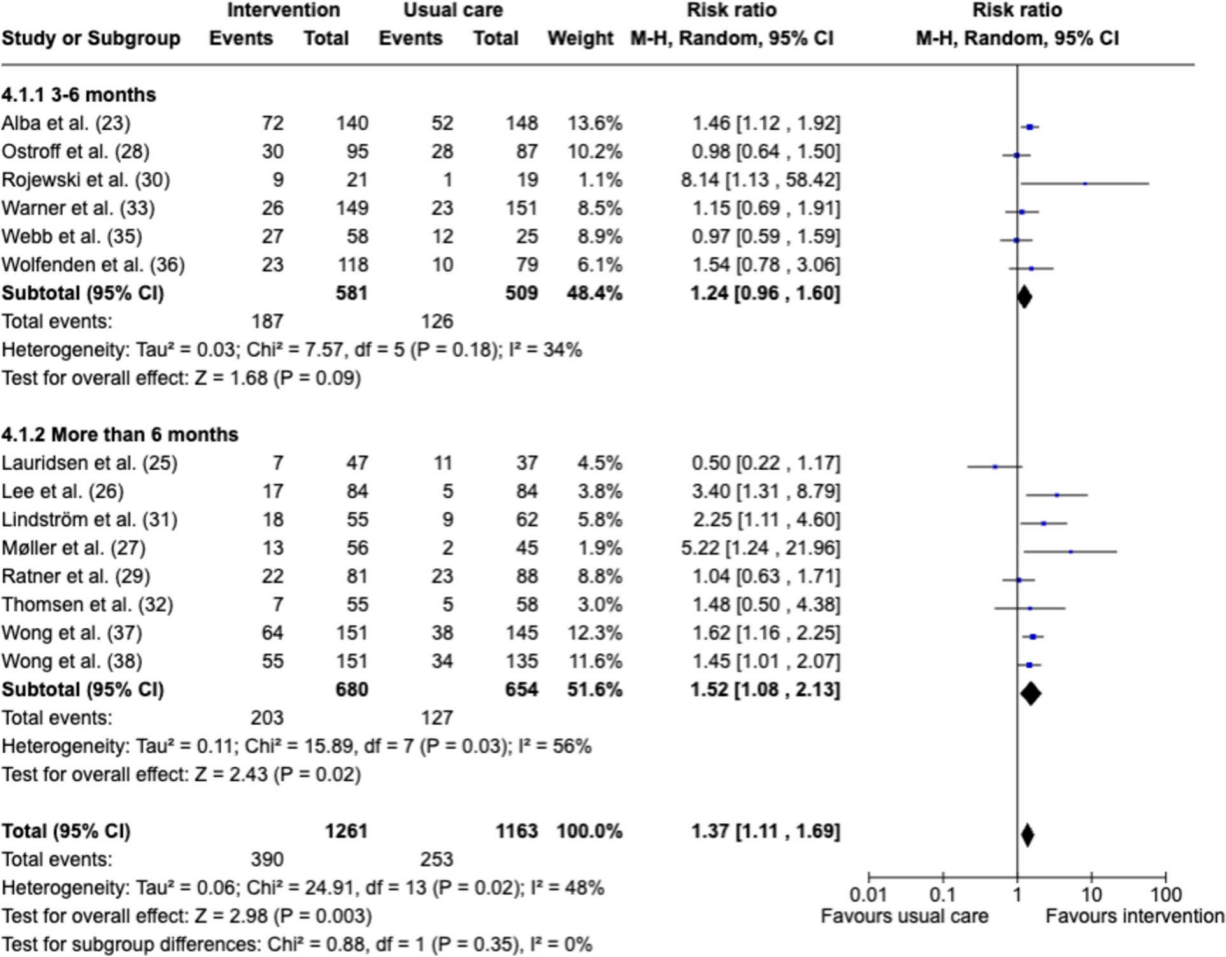
Forest plots for smoking cessation at the duration of follow-up. MH—Mantel–Haenszel random effects model. Risk of bias domains: **A** randomization process, **B** deviations from intended interventions, **C** missing outcomes **D** measurement of the outcome, **E** selection of the reported result, **F** overall bias. Green circle—low risk of bias, yellow circle—some concerns about bias, red circle—high risk of bias

We conducted a detailed analysis of smoking cessation interventions with a particular focus on their efficacy across different follow-up durations: 3–6 months and more than 6 months post-intervention (Fig. 5). The results indicated an RR of 1.24 (95%CI 0.96–1.60, *p* = 0.09) for the 3–6-month follow-up period with no statistical significance and with low heterogeneity (*I*2 = 34%) (Alba et al. 2022; Ostroff et al. 2014; Rojewski et al. 2021; Warner et al. 2011; Webb et al. 2022; Wolfenden et al. 2005). In contrast, for follow-ups of more than 6 months, the RR increased to 1.52 (95%CI 1.08–2.13, *p* = 0.02), indicating a statistically significant improvement in smoking cessation rates compared with a shorter follow-up period (Lauridsen et al. 2022; Lee et al. 2013; Møller et al. 2002; Ratner et al. 2004; Lindström et al. 2008; Thomsen et al. 2010; Wong et al. 2017; Wong et al. 2012). This longer follow-up period demonstrated moderate heterogeneity (*I*2 = 56%).

## Discussion

Our meta-analysis revealed that patients who attend preoperative smoking cessation interventions are more likely to quit smoking at the time of surgery and to maintain abstinence until the last follow-up compared with patients assigned to usual care. The subgroup analysis indicated that the effect of the interventions was larger for intensive interventions compared with brief interventions but that caution should be exercised in the interpretation of these data on intensive interventions, as they were derived from two small studies, and estimates were imprecise. The intensity classification of smoking cessation interventions was from the Cochrane review conducted in 2014 (Thomsen 2014). The classification was applicable to our review, aligning with the World Health Organization (WHO) recommendation that quitting smoking approximately 4 weeks prior to surgery would reduce the risk of complications, and it should comprise multi-behavioral sessions as recommended by NICE (National Institute for Health and Care Excellence 2021; World Health Organization W 2020).

There was a statistically significant improvement in the smoking cessation rate at over 6 months in contrast to no statistically significant result for the period of 3–6 months. This could be related to the fact that all the studies with longer follow-up (> 6 months) used behavior support sessions combined with pharmacotherapy, and all the intensive intervention studies were in the same group. On the other hand, half of the studies with a shorter follow-up period (3–6 months) used the brief advice technique or written information in a brochure without behavioral support sessions. Therefore, it is not clear if the effectiveness is because of longer follow-ups or because of the type of intervention. Behavioral support sessions may offer strategies for managing cravings and provide encouragement to lower the risk of relapse, as 60% of surgical patients who relapse did so during the first 2 months (Webb et al. 2022; Harrogate et al. 2023). Longer follow-ups, even when limited to data collection, may provide a sense of accountability to patients. The fact that their smoking status is being monitored may motivate them to maintain their abstinence and might increase the possibility of exposing the participants to positive reinforcement and skills training (England NHS 2024). Scheduled follow-ups may be important in providing continuous motivation and reinforcement of the necessary skills for successful smoking cessation.

Our findings were similar to the findings of the Cochrane review conducted in 2014 (Thomsen 2014) in terms of smoking cessation at surgical time, the last follow-up, and post-operative complications. However, we have included all RCTs and non-RCTs, which means the findings of our review are more comprehensive. Despite the long period between the two reviews, there were no new intensive smoking cessation interventions. This might be related to the fact that implementing 4 weeks of preoperative face-to-face sessions is challenging in surgical settings where the time between preoperative appointments and surgery is limited and variable. The significant time and resource investment required to deliver smoking cessation may also present a barrier. Surgical and perioperative staff will require training to advise and provide smoking cessation support and time allocated in the pre-operative setting to deliver this support. Moreover, our findings were also similar to a recent review on perioperative smoking cessation interventions at the time of surgery and 12-month follow-up (Warner David and Warltier 2006). However, we have conducted further analysis of smoking cessation interventions with a focus on their efficacy across different follow-up durations to capture all studies with follow-ups of different lengths.

Patients who are scheduled for surgery may often be advised by a healthcare professional (HCP) to quit smoking. Our results show that most of the studies on smoking cessation interventions were delivered by HCPs (Tables 1 and 2). Therefore, preoperative clinical visits present an excellent opportunity to introduce smoking cessation, as, in elective surgeries, patients usually have sufficient time to quit before their surgery. Some patients might be on a long waiting list, and failing to introduce quitting for surgical smokers could represent a missed opportunity. In recent NHS Referral to Treatment (RTT) waiting times data, the shortest waiting times for elderly medicine and cardiothoracic surgery services were 8.49 and 9.06 weeks respectively, while the longest waiting times for oral surgery and ENT services were 19.09 and 18.89 respectively (An and Wong 2020). Even in emergency surgeries, where the time before surgery is limited, quitting smoking at any time before the operation is beneficial because it may reduce the risk of some outcomes (Cooley et al. 2009). The preoperative period could serve as a “teachable moment,” a time when an individual becomes motivated by a health experience to reduce risky behavior (Wong et al. 2012). Therefore, smoking cessation support can be part of preoperative care and may continue in terms of behavioral support and follow-ups postoperatively. There is potential for smoking cessation to be integrated into surgical pathways to increase the engagement of patients and potentially also increase its chances of success (Nohlert et al. 2013).

Our review has some limitations. Most of our included studies were rated as having a high risk of bias overall, mainly due to missing data from participants who dropped out or were lost to follow-up. This issue is a significant challenge in smoking cessation studies, which face a high dropout rate (Ratner et al. 2004; Rojewski et al. 2021; Warner et al. 2011; Webb et al. 2022) and enrolment difficulties (Lauridsen et al. 2022; Lindström et al. 2008; Webb et al. 2022), affecting the reliability and generalizability of the findings. Further investigation, including a qualitative approach to smoking cessation interventions, is needed to explore the facilitators and barriers, particularly regarding patients who decline to participate in the intervention or who withdraw. This approach could potentially ensure the interventions’ acceptability to targeted patients, thereby reducing dropout rates and missing data.

Bias due to inadequate outcome assessment was also common in this review due to studies relying on self-reported smoking cessation instead of biochemically verified outcomes. Outcomes were considered to have “some risk of bias” if urine or saliva tests were mailed to participants, due to the lack of blinding in the trials. Moreover, this approach to verifying results remotely was susceptible to tampering. Another significant limitation is the diversity in outcome measurement in the included studies. Despite the effort to use standardized outcome measurements for smoking cessation studies, our review indicates that this is still not widely implemented (West et al. 2005). Despite the small difference between validated and non-validated self-reported smoking cessation and the resources needed to biochemically validate smoking cessation, using a verified and standardized smoking cessation verification tool would reduce the risk of bias. Another source of high heterogeneity was due to the patient selection. Some studies recruited patients from one type of surgery and others from a mix of elective surgeries.

## Conclusion

In our review, preoperative smoking cessation interventions increase the quit rate of smoking at surgery time and continuous quitting to the last follow-up, and there was no evidence of a difference in postoperative complications. Intensive smoking cessation interventions are likely to be effective in terms of quitting rates and reducing postoperative complications; however, the available data are limited. The use of standardized outcome measurements is recommended to reduce heterogeneity. Further studies are needed to examine up-to-date smoking cessation support with better integration within the surgical pathways. There is also a need for further investigation focusing on patient perspectives to address low enrolment and high dropout rates from smoking cessation interventions.

## Supplementary Information

Additional file 1: Appendix 1: Search strategy. Appendix 2: Data extracting form. Appendix 3: Risk of bias assessment for RCTs. Appendix 4: Quality assessment for non RCTs

## Data Availability

No datasets were generated or analysed during the current study.

## Abbreviations

NICE: National Institute for Health and Care Excellence
BTS: The British Thoracic Society
PICO: Population, Intervention, Comparator, Outcome
POMS: Postoperative Morbidity Survey
CINAHL: Cumulative Index to Nursing and Allied Health Literature
PRISMA-S: Preferred Reporting Items for Systematic reviews and Meta-Analyses Statements
TAG: TAG Cochrane Tobacco Addiction Group
ROB: Risk of bias
NIH: National Institution of Health
RCT: Randomized control trial
RR: Relative risk
CI: Confidence interval
WHO: World Health Organization
HCP: Healthcare professional
RTT: Referral to treatment
