# Supplementary material for: Preoperative smoking cessation interventions: a systematic review and meta-analysis
Source: Perioper Med (Lond). 2025 Jan 10;14:5. doi: 10.1186/s13741-024-00479-4 (PMC11724455; doi:10.1186/s13741-024-00479-4)

Appendix A: Search strategy

| Concept | CINAHL (119) | | Medline (130) | | EMBASE (135) | | Web of Science (1,935) | | Cochrane Library (49) | |
| --- | --- | --- | --- | --- | --- | --- | --- | --- | --- | --- |
|  | MH Terms | Keywords | MeSH terms | Keywords | EMTREE terms | Keywords | Topic terms | Keywords | MeSH descriptor | Keywords |
| 1. Preoperative | 1. Surgical patients  2. Preoperative period | 1. Surgical patient*  2. operative patient* 3. preoperative patient* 4. preoperative patient* 5. post-operative patient* 6. postoperative patient* 7. perioperative patient* | 1. exp preoperative period | 1. surgical patient*.mp 2. preoperative patient*.mp.  3. pre-operative patient*.m 4. operative patient*.mp 5. Postoperative patient*.mp.6. post-operative patient*.mp 7. perioperative patient*.mp. | 1. exp surgical patient | 1. preoperative patient*.mp. 2. pre-operative patient*.mp. 3. operative patient*.mp. 4. postoperative patient*.mp. 5. post-operative patient*.mp. 6. perioperative patient*.mp. | 1. TS=(surgical patient*) 2. TS=(Preoperative*) 3. TS=(Pre-operative*) 4. TS=(Perioperative*) | (ALL=(Surgical patient*)) OR ALL=(preoperative patient*)) OR ALL=(Pre-operative patient*)) OR ALL=(Postoperative patient*)) OR ALL=(Post-operative patient*)) OR ALL=(Operative patient*)) OR ALL=(Perioperative patient*) | MeSH descriptor: [Preoperative Period] | (Surgical patient*) 2. (operative patient*) 3. (pre-operative patient* OR preoperative patient*) 4. (post-operative patient* OR postoperative patient*) 5. (perioperative patient*) |
| 2. Smoking | 1. Smoking  2. Tobacco products | 1. Smoking 2. Nicotine 3. Tobacco 4. Cigar 5. Ecigarette 6. E-cigarette 7. Electronic nicotine delivery systems 8. Electronic cigarette 9. Vaporizer 10. Ecig 11. Vaping 12. juuling | 1. exp Smoking 2. exp Tobacco smoking | 1 Tobacco Smoking 2. Cigar Smoking 3. Pipe Smoking 4. Smoking Devices 5. Cigarette Smoking 6. Water Pipe Smoking 7. Smoking.mp. | 1.. exp Smoking | 1. smoking.mp. 2. water pipe smoking.mp. 3. cigarette smoking.mp. 4. cigar smoking.mp. 5. tobacco smoke.mp. 6. Ecigarette.mp. 7. E-cigarette.mp. 8. Tobacco consumption.mp. | 1. TS=(smoking) 2. TS=(tobacco) 3. TS=(cigar) | ((ALL=(Smoking )) OR ALL=(nicotine)) OR ALL=(tobacco)) OR ALL=(cigar)) OR ALL=(ecigarette)) OR ALL=(e-cigarette )) OR ALL=(electronic nicotine delivery systems)) OR ALL=(electronic cigarette)) OR ALL=(vaporiser)) OR ALL=(ecig)) OR ALL=(vaping)) OR ALL=(juuling). | 1. MeSH descriptor: [Smoking] 2. MeSH descriptor: [Tobacco] 3. MeSH descriptor: [Electronic Nicotine Delivery Systems] | 1. (Smoking) 2. (nicotine) 3. (tobacco) 4. (cigar) 5. (ecigarette OR e-cigarette) 6. (electronic cigarette) 7. (vaporizer) 8. (ecig) 9. (vaping) 10. (juuling) |
| 3. Cessation | 1. smoking cessation  2. Tobacco use cessation products | 1. cessation 2. quit* 3. stop* 4. give up 5. refrain” | 1. exp smoking cessation 2. exp Tobacco smoking cessation | 1. Tobacco Use Cessation.mp 2. Smoking Cessation.mp 3. Tobacco Use Cessation Devices.mp. | 1. exp Smoking cessation program 2. exp Smoking cessation. | 1. cessation.mp. 2. quit*.mp. 3. stop*.mp. 4. give up.mp. 5. Refrain.mp | 1. TS=(Cessation) 2. TS=(quit*) 3.TS=(stop). | (ALL=(cessation)) OR ALL=(quit* )) OR ALL=(stop*)) OR ALL=(give up)) OR ALL=(refrain ) | 1. MeSH descriptor: [Smoking Cessation] | 1. (cessation) 2. (quit*) 3. (stop*) 4. (give up) 5. (refrain) |

Appendix B: Data extracting form


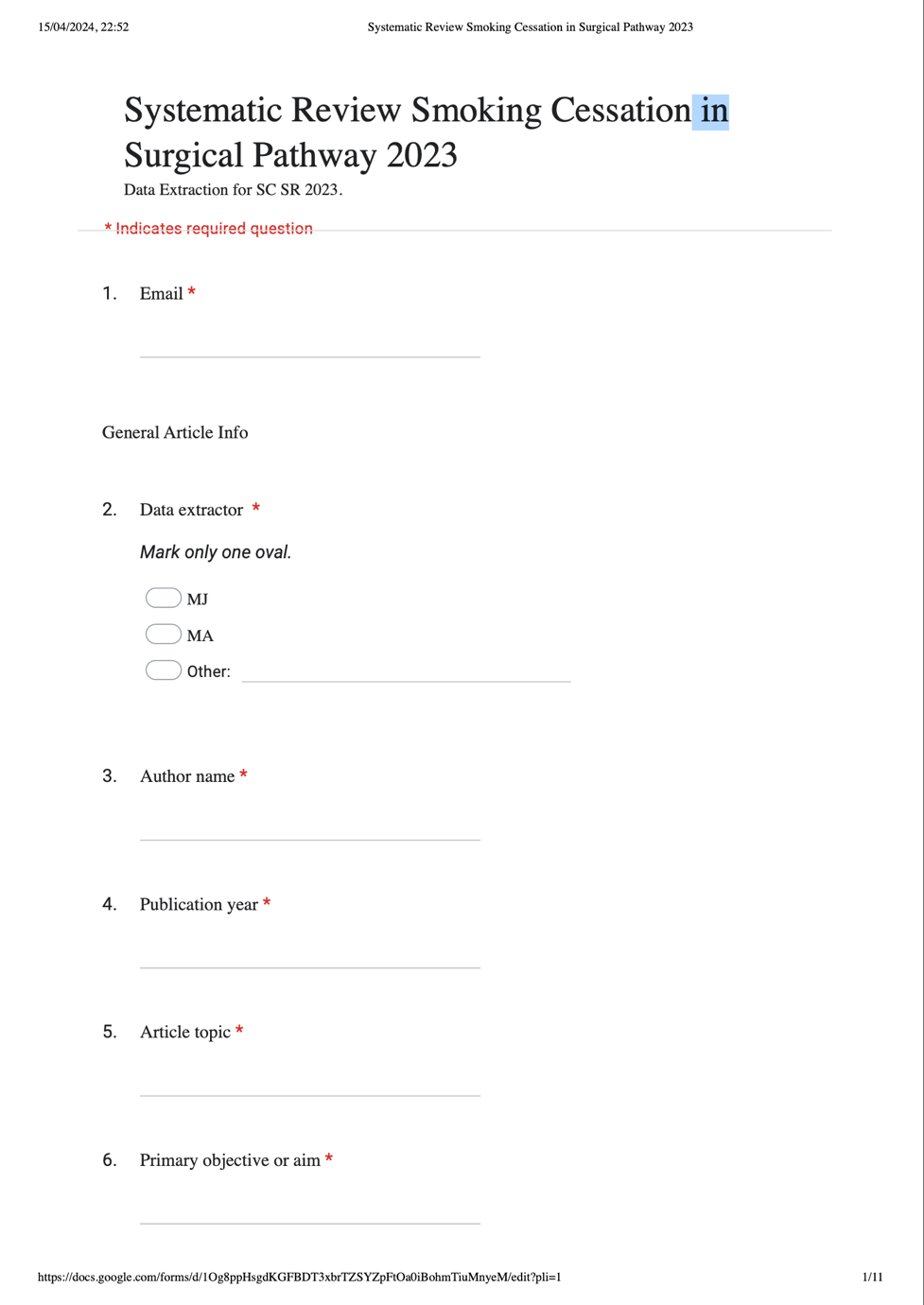


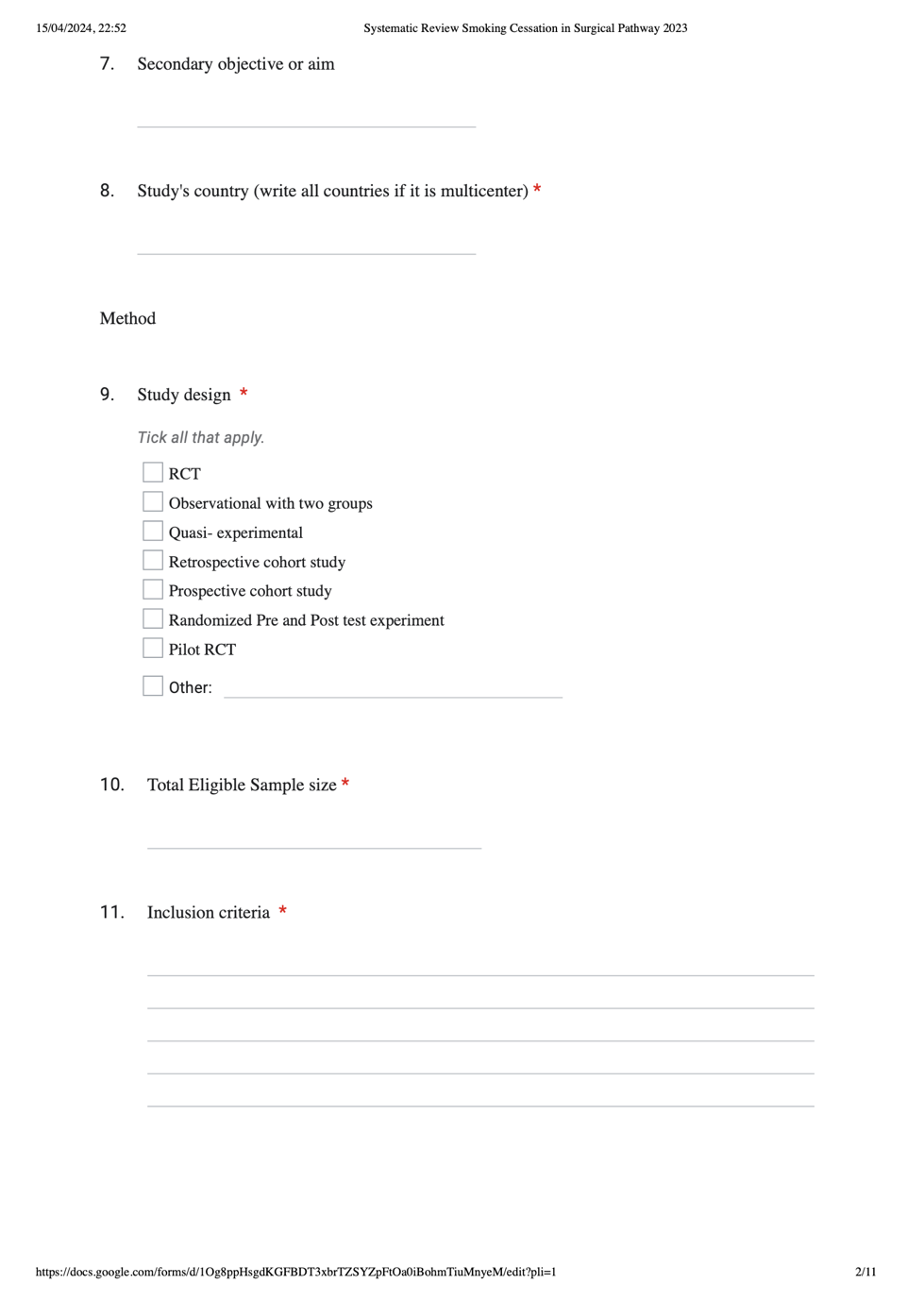

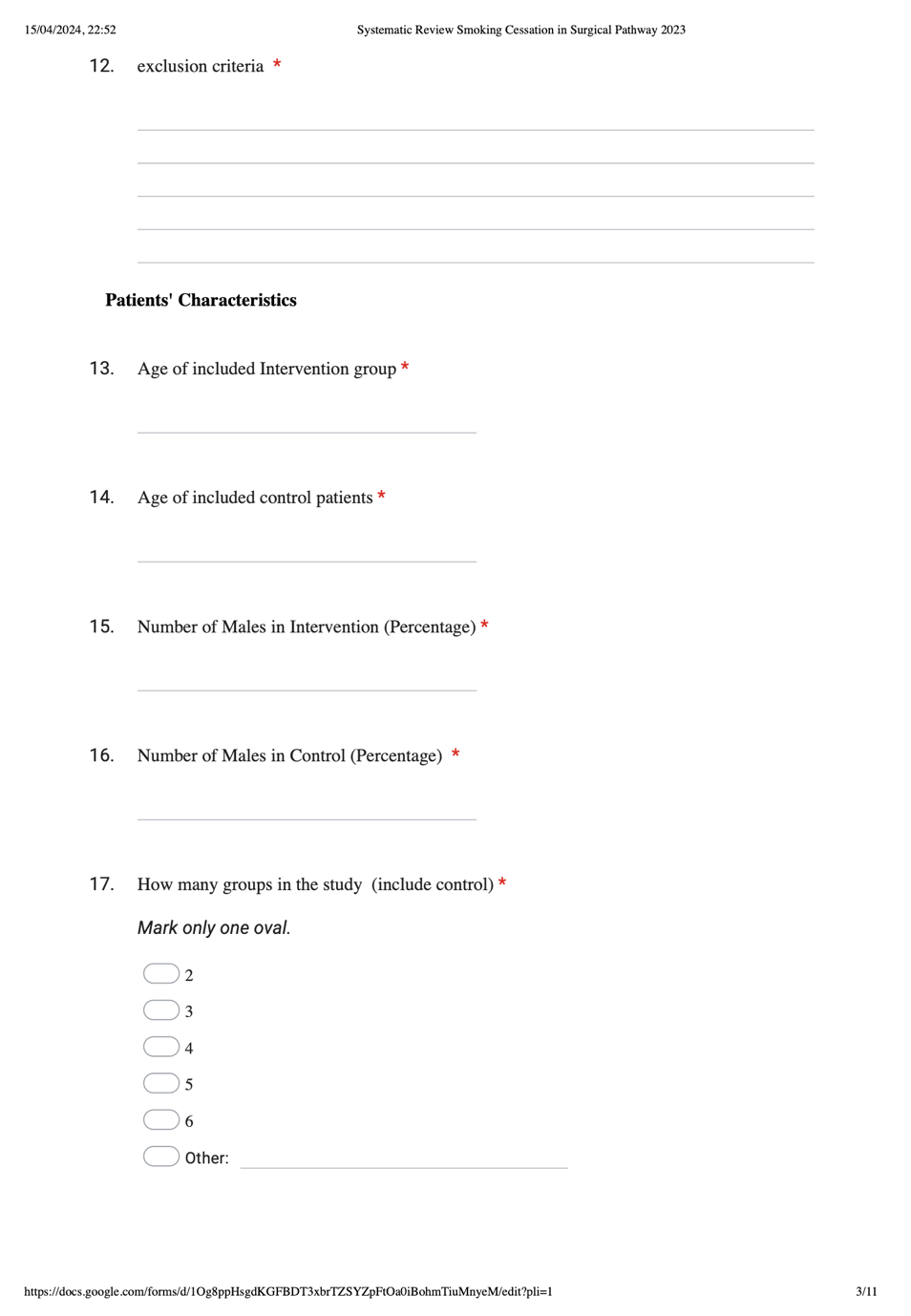


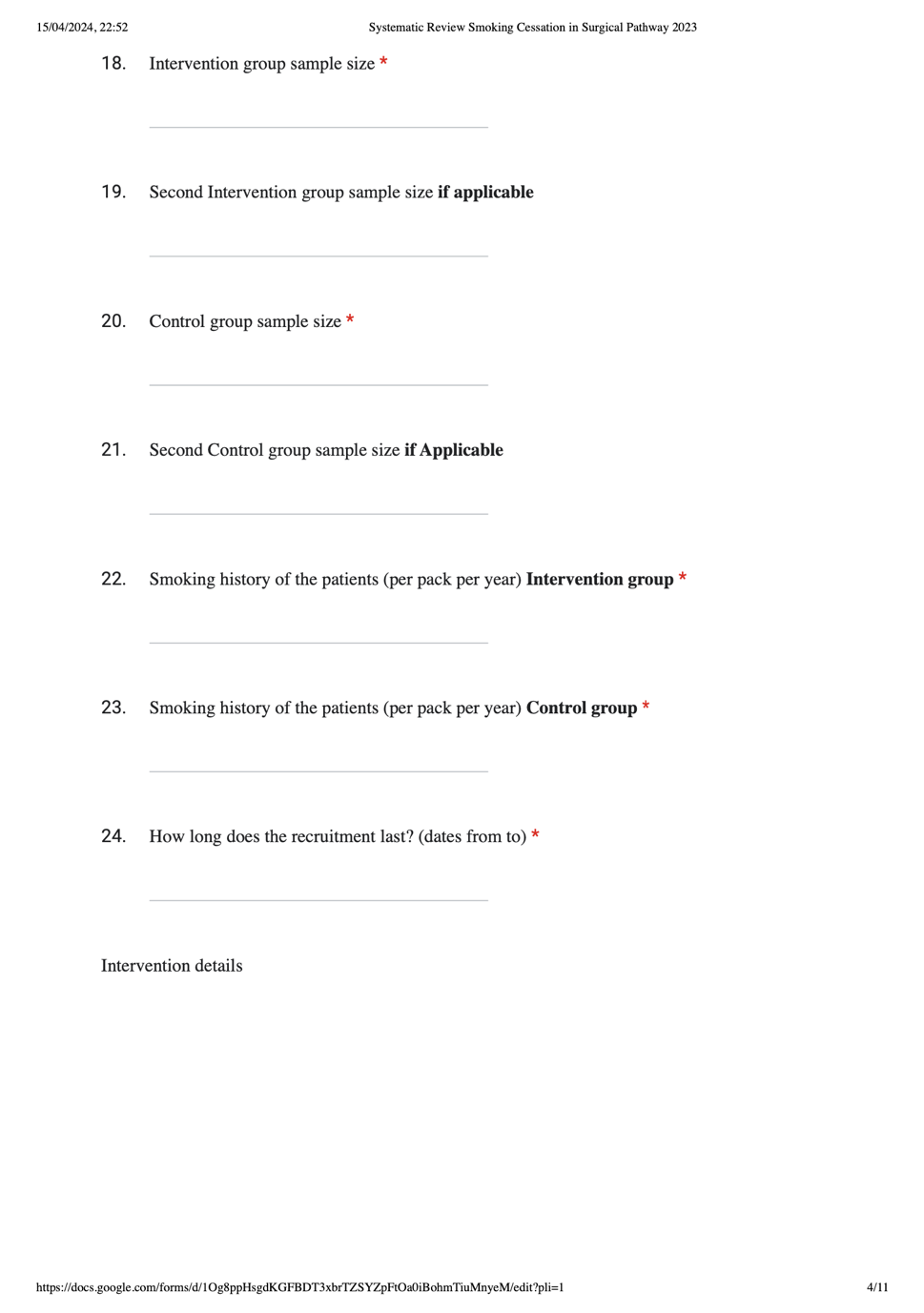

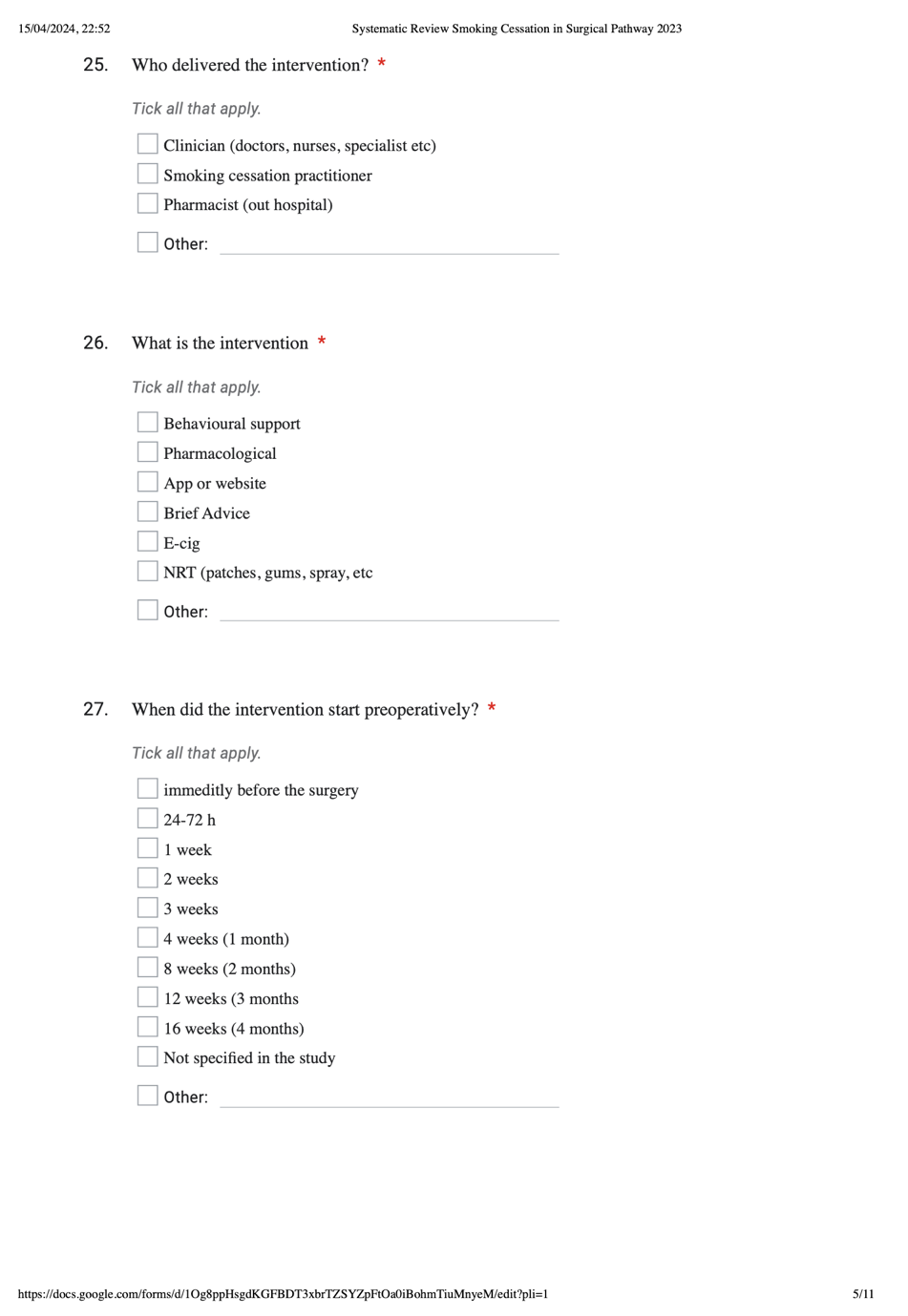


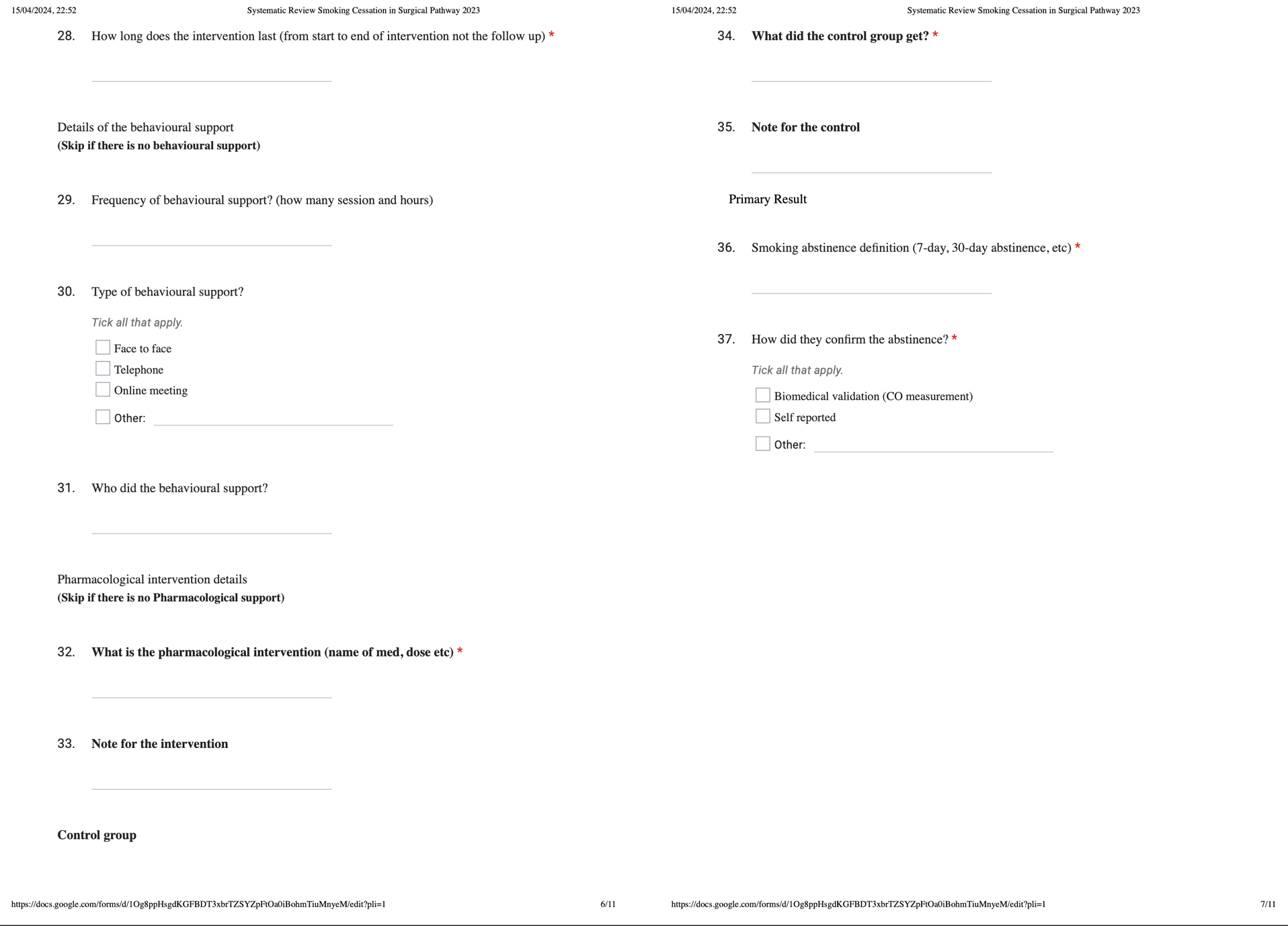


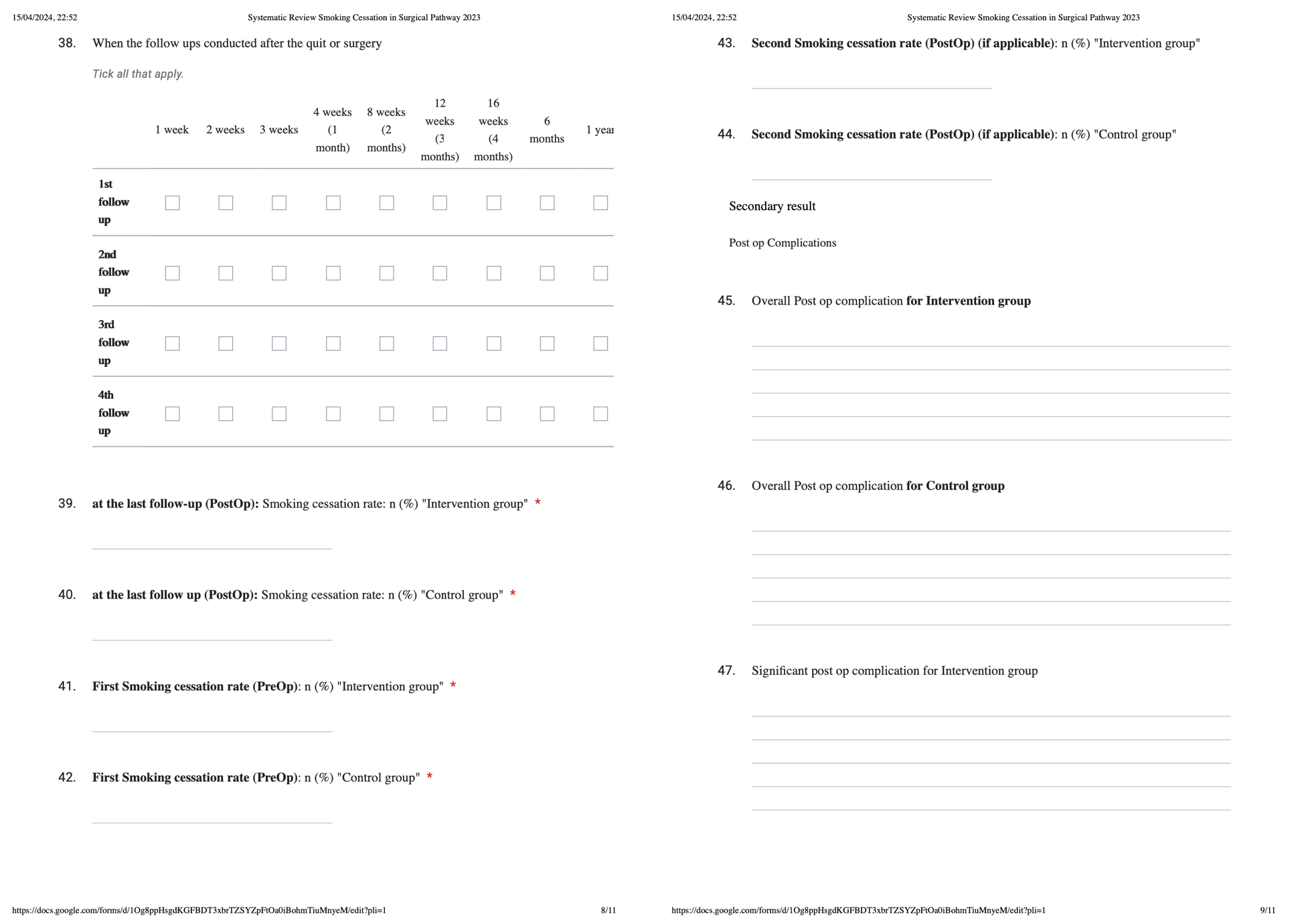


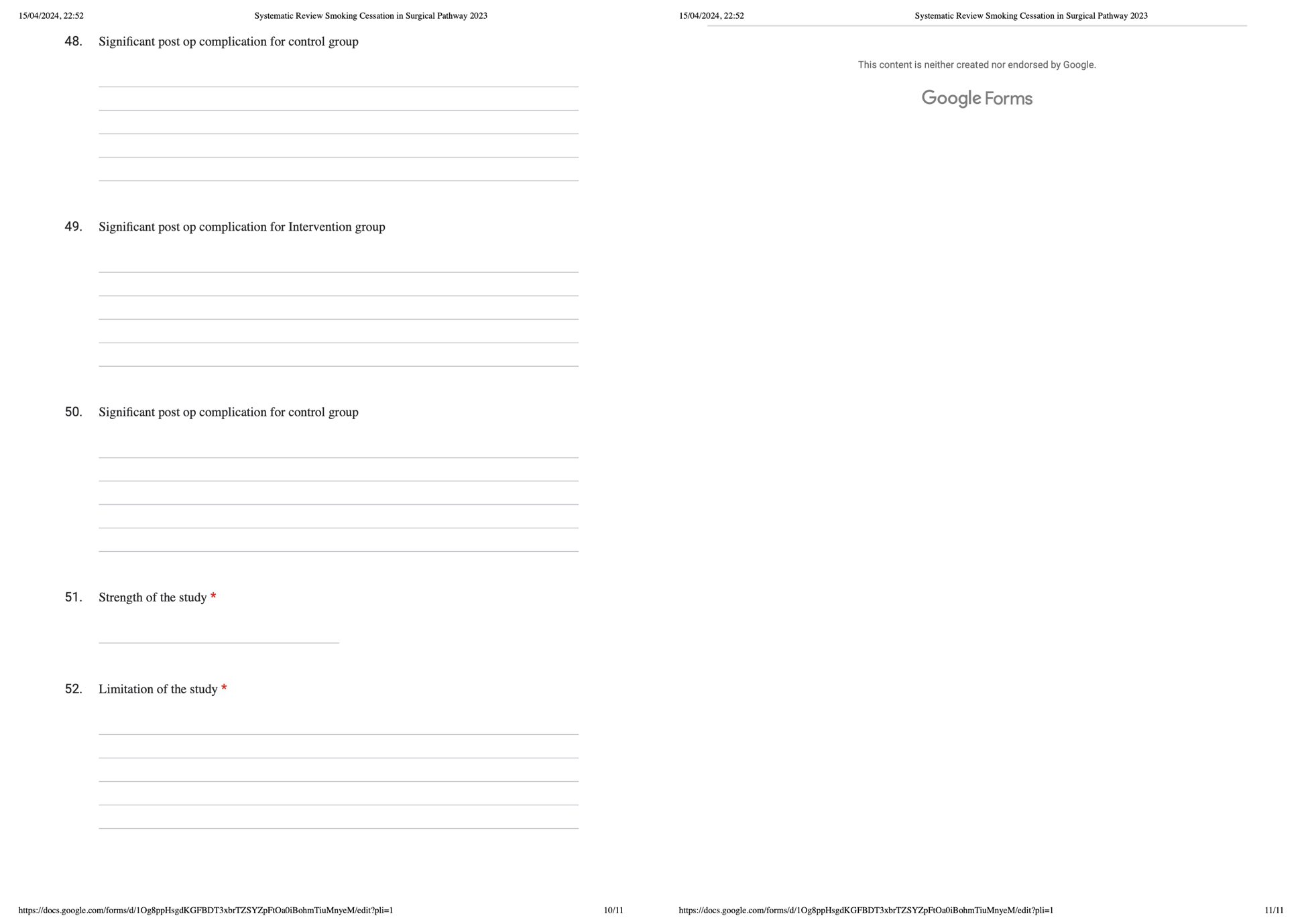


Appendix C: Risk of bias assessment for RCTs


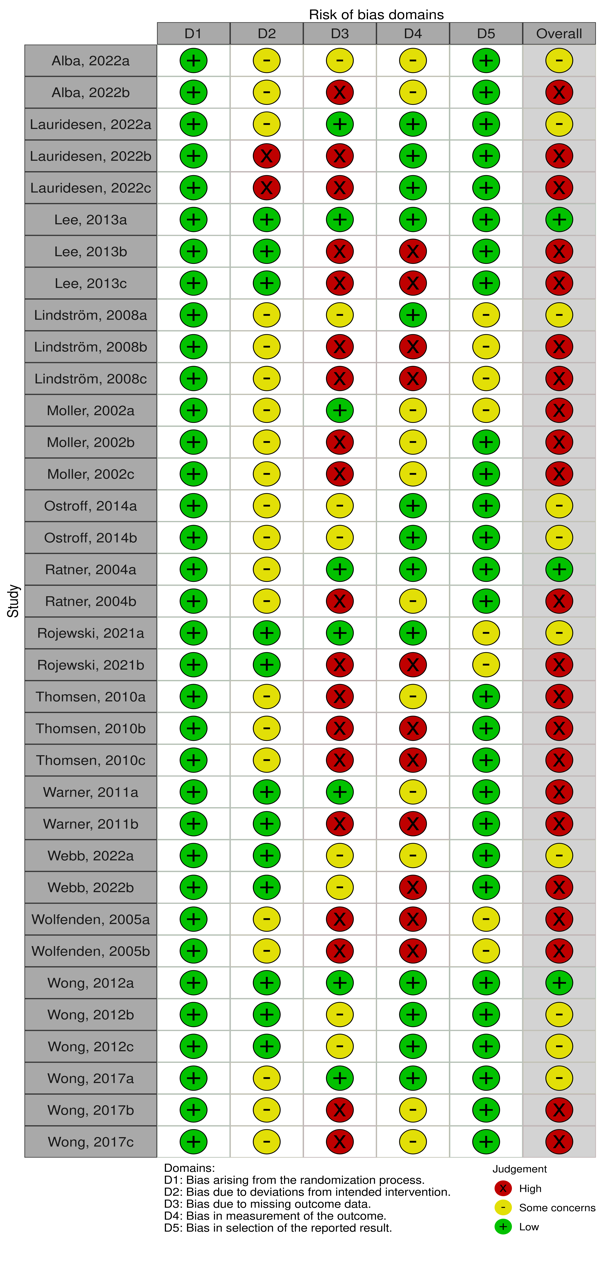


A: Smoking cessation at surgery time outcomes

B: Smoking cessation at the last follow-up outcomes

C: Postoperative complication outcomes

Appendix D: Quality assessment for non RCTs:


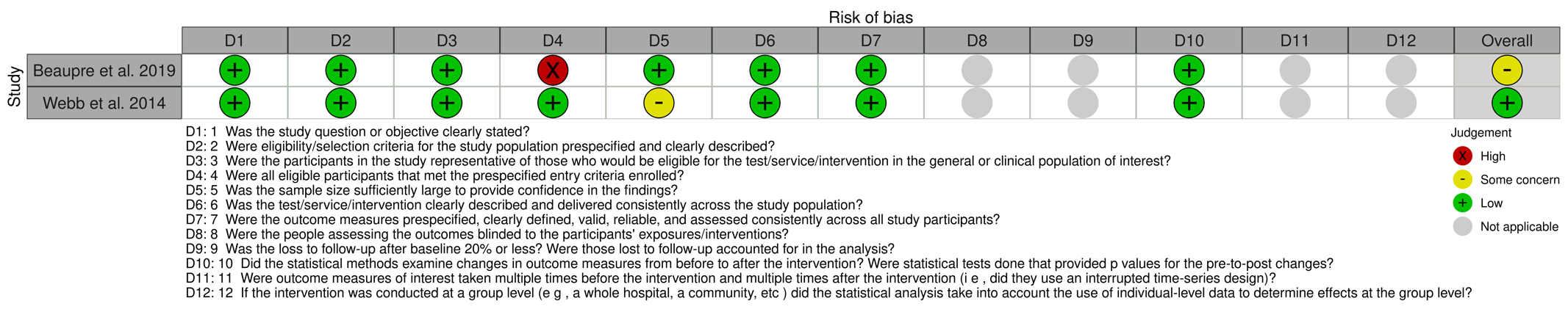

Supplement: Supplementary file 1 — Additional file 1: Appendix 1: Search strategy. Appendix 2: Data extracting form. Appendix 3: Risk of bias assessment for RCTs. Appendix 4: Quality assessment for non RCTs [file 13741_2024_479_MOESM1_ESM.docx]
